# Supplementary material for: Computational anatomy: the cerebellar microzone computation
Source: Oxf Open Neurosci. 2025 May 16;4:kvaf001. doi: 10.1093/oons/kvaf001 (PMC12089033; doi:10.1093/oons/kvaf001)
Supplement: Review_history_for_OXFNSC-2024-010_kvaf001_R2_kvaf001_odt [file review_history_for_oxfnsc-2024-010_kvaf001_r2_kvaf001_odt.zip › Review_history_for_OXFNSC-2024-010_kvaf001.R2_kvaf001.odt]

Review history for OXFNSC-2024-010.R2

Round 1 reviews
Reviewer 1
Recommendation - Reject
Comments to the Author
Major comments:
1. The claims proposed in this manuscript are in contradict with the current, accepted understanding of the cerebellum, namely the Marr-Albus-Ito model. The authors need to demonstrate that the proposed model can explain some experiments results that cannot be explained by the Marr-Albus-Ito model.
2. The manuscript, in the current form, is too long, so potential readers will find it difficult to follow the author's reasoning. I recommend the authors to reduce the text length and focus on the most important claim.
3. I do not understand the results of the model simulations. There is no comparison with physiological data.
4. While the manuscript is lengthy, the important details of model simulations are missing. What single-neuron models are used in these simulations? After reading this manuscript, I do not think that I can reproduce the same simulations by myself. Please describe the simulation details.
Minor comments:
- Abstract: The sentence "It is poorly understood how the cerebellum codes and processes information" is an overstatement. The cerebellum is the most well-understood area among the central nervous system in anatomy and computation. 

Reviewer 2
Recommendation – Minor Revision
Major comments:
Even though I am inclined to agree with the author that a large portion of the explanation of cerebellar function can be attributed to direct consequences of wiring, particularly in simpler organisms such as swimmers, there are many functions that cannot be explained without recourse to cerebellar dependent sensorimotor adaptation (examples abound in bipedal postural research, tool use, motor skill acquisition).
Also, the paper has some bombastic categorical statements (e.g., "i.e., not understood", which misses nuance in published literature, particularly discussion sessions of earlier papers by Rodolfo Llinas and other contemporaries. Kazantsev and collaborators have published a control system based on phases of inferior olive oscillations which strongly resemble the innate system proposed in the paper). In addition, while true that there are many problems/dogmatic thinking within the current picture of cerebellar function, many will agree that population coding at the level of the microzone is the main output of the cerebellum. And that this output is functional even before being learning. In fact, many researchers think that cerebellar learning happens 'on top' of a system that has been pre-wired for function.
I feel strongly that the paper would benefit from a more nuanced appraisal of previous thought on cerebellar innate functions, and a discussion of skill acquisition and adaptation, which still provably happen.
Other comments:
Figure 3 needs better aspect ratios and can be better summarized in a simple transfer function (e.g., phase vs rate).
Figure 4 Information needs to be better summarized. It would be convenient to show an I-O parameter space.

Decision letter
Manuscript ID OXFNSC-2024-010 entitled "Computational anatomy: the cerebellar microzone computation" which you submitted to the Oxford Open Neuroscience, has been reviewed.  The comments of the reviewer(s) are included at the bottom of this letter.

Please note that this journal operates with transparent peer review. This means that if your submission is accepted for publication, the full peer review history of your article will publish online alongside your article. This includes reviewer comments, editor decision letters, and your author responses.

Based on the reviewer comments, I am inviting you to submit a manuscript with major revisions. I invite you to respond to the reviewer(s)' comments and revise your manuscript within the next 60 days from today date.

Please consider all of the reviewer comments carefully. I encourage you to focus on providing:

- further acknowledgement of the limitations of your approach to cerebellar function

- a more nuanced appraisal of previous work

- a substantial rewrite, to avoid colloquial or 'bombastic' language and improve clarity

- further details of your model simulations

To revise your manuscript, log into https://mc.manuscriptcentral.com/oxfnsc and enter your Author Centre, where you will find your manuscript title listed under "Manuscripts with Decisions."  Under "Actions," click on "Create a Revision."  Your manuscript number has been appended to denote a revision.

You may also click the below link to start the revision process (or continue the process if you have already started your revision) for your manuscript. If you use the below link you will not be required to login to ScholarOne Manuscripts.

*** PLEASE NOTE: This is a two-step process. After clicking on the link, you will be directed to a webpage to confirm. ***

https://mc.manuscriptcentral.com/oxfnsc?URL_MASK=1caec93c651f49b0b3973e9b513a9d2c

You will be unable to make your revisions on the originally submitted version of the manuscript.  Instead, revise your manuscript using a word processing program and save it on your computer.  Please also highlight the changes to your manuscript within the document by using the track changes mode in MS Word or by using bold or colored text.

Once the revised manuscript is prepared, you can upload it and submit it through your Author Centre.

When submitting your revised manuscript, you will be able to respond to the comments made by the reviewer(s) in the space provided.  You can use this space to document any changes you make to the original manuscript.  In order to expedite the processing of the revised manuscript, please be as specific as possible in your response to the reviewer(s).

IMPORTANT:  Your original files are available to you when you upload your revised manuscript.  Please delete any redundant files before completing the submission.

Because we are trying to facilitate timely publication of manuscripts submitted to the Oxford Open Neuroscience, your revised manuscript should be uploaded within the next 60 days from today date.  If it is not possible for you to submit your revision in a reasonable amount of time, we may have to consider your paper as a new submission.

Once again, thank you for submitting your manuscript to the Oxford Open Neuroscience and I look forward to receiving your revision.

Authors’ response
We thank both reviewers for their constructive comments. We have interleaved our responses.

REVIEWER: 1

Comments to the Author
Major comments:

Comment
1. The claims proposed in this manuscript are in contradict with the current, accepted understanding of the cerebellum, namely the Marr-Albus-Ito model. The authors need to demonstrate that the proposed model can explain some experiments results that cannot be explained by the Marr-Albus-Ito model.

Response
We attempt to explain detailed neuroanatomy in the light of evidence of linear signalling. We believe it is difficult to reconcile evidence of linear signalling with evidence of multi-site synaptic plasticity. Our claims are an attempt to find an alternative to the traditional understanding of the cerebellum which explains anatomy as functional and accounts for linear signalling by proposing that anatomy can be explained as a network of linear units. We now summarise strengths and weaknesses of the ability of the model to account for the evidence in the two right-hand columns of Figure 2.

Evidence of anatomy is rich and generally well established. We argue it is functionally more significant than simply providing a way to move information around. In this paper, we focus on the microzone computation. We describe the basis in anatomy for derivation of model parameters in section 4. We also cite in that section the relevant evidence for linear transmission. This paper builds on a model of the granular layer (in press). Evidence of linear transmission in the granular layer is rich, although it remains contentious. Supplementary materials contain a review of the evidence which is adapted from the granular layer paper.

Comment
2. The manuscript, in the current form, is too long, so potential readers will find it difficult to follow the author's reasoning. I recommend the authors to reduce the text length and focus on the most important claim.

Response.
We agree that short is good if that is possible. We have reduced the length of the manuscript from 8,370 words to 7,300 words despite adding a new section (the new Discussion, 630 words) requested by reviewer 2. Unfortunately, the nature of the subject means it is not possible to prioritise the ideas without losing the rationale. There is no overarching core proposition. We understand and agree with the value of prioritising, but the ideas are not a hierarchy.

To expand on that:

A. We challenge what, as you say, is for some researchers the accepted understanding of the cerebellum. While this presents an exciting opportunity, it comes with the problem that we must present the ideas and methods from scratch, because we do not build on an existing understanding, or on existing models.

B. Our methodological approach is uncommon. We infer a mechanism from the evidence and then infer function from the mechanism. Our claim is to explicitly relate unit functions to the physical hardware of the system, for which there is a good case in neuroanatomical evidence, and evidence for linear communication. To describe that from a standing start is challenging. Unfortunately, there are no shortcuts. We can’t allude to a common understanding that the reader already has, or a consensus. The computational element is not mathematically challenging but equally it is not reducible to an algorithm or mathematical shorthand.

C. It is important to visualise the network at a detailed level because we argue that information has the spatial dimensions. A knowledge of neuroanatomy is necessary to understand the derivation of model parameters. A schematic is not sufficient. We must therefore describe neuroanatomy for the general neuroscience reader.

D. We do not benefit from publishing conventions. We mean, the content does not fit the conventional structure either of experimental or computational papers. In some ways, speaking generally, experimental and computational work proceeds separately, each with their own literature. Part of our motivation was to bridge disciplines, but that comes with the problem that we can’t use – for example – the usual headings to help the reader navigate.

E. One way to reduce the length of the paper would be to omit the final section, section 5 (which used to be the Discussion), and/or the new Discussion (now about motor learning, requested by reviewer 2). We feel the reader may naturally wonder about the functional context for the network computation, and how the output of functionally related networks is coordinated. Section 5 and the new Discussion are intended to provide context. That said, we are happy for this (whether to retain section 5 and/or the Discussion section) to be a reviewer/editor decision.

Comment
3. I do not understand the results of the model simulations. There is no comparison with physiological data.

Response
Our data represent information (rather than biophysical properties) coded in firing rates or dendritic/somatic signals, on a normalised scale. This is of course standard for axonal signals, where it is accepted that firing rates code a value, but an innovation for dendritic signals (which we argue conserve rate information).

Speaking generally, rate information transitions through multiple biophysical steps even in transit through a single neuron. Normally, these are measured on different scales. Scales are arbitrary per se but bring consistency to data collection. We float the idea that on a normalised scale, the data coded at each step are related by straightforward functions, so that they can provide the physiological form of mathematical operations, and therefore add steps (but not necessarily sophistication) to neural computations. This is counter to the appearance of the hard-to-see relationship of biophysical measurements. However, it is consistent with evidence (which we cite) that rate information is linearly conserved in communication between cerebellar cells. If we take this step, there is – we argue – a computational effect of ensemble anatomy.

The evidence we aim to explain is anatomy and linear signalling. The purpose of the computer simulation is to populate the hypothesis with physiological parameters to test the proposal that known anatomy has, plausibly, a computational effect. Unit functions can be expressed as C⟨i⟩, where ⟨i⟩ is the mean of input values (the sum also works) and C is a constant which depends on the unit type. C is unknown but unnecessary to demonstrates the statistical effects we claim anatomy may have. On a related note, in physiological practice it is unnecessary for C to be precisely genotypically coded at each step in the computation because learning provides a microzone-wide gain adjustment. In this contention, learning adjusts the gain of the anatomically-mediated unlearned network computation. Learning is the subject of the revised Discussion.

Comment
4. While the manuscript is lengthy, the important details of model simulations are missing. What single-neuron models are used in these simulations? After reading this manuscript, I do not think that I can reproduce the same simulations by myself. Please describe the simulation details.

Response
We do not use (or adapt) existing neuron models. We see anatomy as the physical form of functionally-discrete populations of computational units. Unit functions (the computation performed by a unit) are linear. The function depends on the unit type. Units are physically diverse. Stellate cells, glomeruli and Purkinje cell dendritic compartments are all units, for example. The size and shape of unit populations are defined by the topography of inputs to the cerebellum. We propose the granular layer is divided functionally into strips – ‘ranks’ – mirroring microzonal organisation. Units in a topographically defined region – i.e., in a rank or in a microzone – are intermingled but organised functionally into layers. While a single unit receives anatomically fixed inputs, wiring of the system has the computational result that single units effectively randomly sample the whole population of the outputs of the afferent layer. The network is thus able to exploit statistical effects of iterative random sampling. Since anatomy is generally well known, we can replicate physiology in silico as a layered network of parameters. While this is an abstraction, it is not, computationally speaking, a simplification – at any rate, that is part of our argument.

We now describe this in section 2. The revisions have made this part of the paper better structured and pithier. Of course, it may be wrong that unit functions are linear. Also, we may be wrong about the physical form units have. However, we believe the content and revised structure of the paper should enable the reader to reproduce the microzone computation.

A related issue is that the paper builds on a model of the granular layer computation (in press). The microzone computation, where we focus in the present paper, takes as input the output of that model. The present paper does not include a full methodology of the granular layer model. We address that by including a link to a preprint of the granular layer paper in the reference section, and describing the derivation of mossy fibre rates in supplementary materials. Supplementary materials also include a discussion of the evidence of granular layer linear signalling.

Minor comments:

- Abstract: The sentence "It is poorly understood how the cerebellum codes and processes information" is an overstatement. The cerebellum is the most well-understood area among the central nervous system in anatomy and computation.

Response
This sentence has been deleted.

Closing author remark
We hope our responses and revisions address your comments. We understand your reservations. It took us a while to get used to some of our own conclusions, but we think it stacks up, if we can put it like that. We believe it is valid competition for existing models.


REVIEWER: 2

Comments to the Author
Major comments:

Comment
Even though I am inclined to agree with the author that a large portion of the explanation of cerebellar function can be attributed to direct consequences of wiring, particularly in simpler organisms such as swimmers, there are many functions that cannot be explained without recourse to cerebellar dependent sensorimotor adaptation (examples abound in bipedal postural research, tool use, motor skill acquisition).

Response
Absolutely. This paper is part of a set of proposals. The nature of the subject means it is not possible to present the ideas in a single paper – there is no pithy algorithm and the proposals can’t be condensed into mathematical shorthand. This involves the challenge of breaking the proposals into smaller, stand-alone parts in order to publish them. The omission of learning from the original draft of the present paper is an example of the process. We agree that it is a better paper with a discussion of learning. In the revised submission, we have added a new section. This has become the new Discussion; the old Discussion has become section 5.

Comment
Also, the paper has some bombastic categorical statements (e.g., "i.e., not understood", which misses nuance in published literature, particularly discussion sessions of earlier papers by Rodolfo Llinas and other contemporaries. Kazantsev and collaborators have published a control system based on phases of inferior olive oscillations which strongly resemble the innate system proposed in the paper). In addition, while true that there are many problems/dogmatic thinking within the current picture of cerebellar function, many will agree that population coding at the level of the microzone is the main output of the cerebellum. And that this output is functional even before being learning. In fact, many researchers think that cerebellar learning happens 'on top' of a system that has been pre-wired for function.

Response
That’s good to hear. We have received some hostile feedback. On the other hand, it has improved the papers – not the mechanics of the model, but high-level interpretation and presentation of the ideas.

About our language, we are sorry. It was not our intention to discount other thinking and we are a little horrified it came across like that.

Comment
I feel strongly that the paper would benefit from a more nuanced appraisal of previous thought on cerebellar innate functions, and a discussion of skill acquisition and adaptation, which still provably happen.

Response
Learning is now the subject of the new Discussion, as we mention above. Our revised approach to appraising other models is to take a step back. We are anxious not to make over-pithy comments about what we (of course) agree is a nuanced subject. Our original thinking was that it might be useful to the general neuroscience reader if the theoretical background was condensed and limited to supervised learning models, which some researchers regard as the accepted understanding of the cerebellum (reviewer 1 makes this point). We have revised that approach. We agree it is misrepresentative; this is more than ever a risk of discussing cerebellar function in a sidebar. The functions of the cerebellum now seem to extend to almost everything, and are still being added to. In addition to cognitive-affective functions, language and a range of psychiatric conditions, it is implicated in the neuro-immune system, regulation of defensive states, the mirror neuron system and social functions. We now limit our discussion to the mechanistic approach we take generally. Modular cerebellar wiring is highly conserved across species. Our interest is in functionality that modules may share, without looking beyond that at how a (perhaps) standard computation is put to different uses. That said, we model swimming as an example of possible context.

Other comments:

Comment
Figure 3 needs better aspect ratios and can be better summarized in a simple transfer function (e.g., phase vs rate).

Response

A. General notes.

While there is a computational element of the paper, it is not primarily a computational paper. In our approach, the simulation is simply the hypothesis populated with parameter values taken from the literature. There is no separation of the hypothesis and the model/mechanism. Our aim/scope is to present a reinterpretation of the evidence, for which the simulation is computational support.

If it was a computational paper, examples might be poor presentation of the ideas. But in our approach, the computer simulation has the role of a test (vs summary). The purpose of the computer work – in the paper generally – is to demonstrate proof of concept. ‘Proof’ does not refer to mathematical proof, but a practical demonstration, using examples.

This relates to our approach. In some network models, the function of the system is a proposal which becomes an assumption of the model/algorithm which solves the problem. We work in reverse – we infer a mechanism from the evidence then infer function from the mechanism. With this approach, we attempt to explain anatomy as functional and account for linear signalling by proposing that anatomy can be explained as a network of linear units. Working this way, the computational work isn’t a mathematical statement of the proposals but a method to test the performance of the system.

An important concern when the ideas were in development was whether system performance was dependent on the range and frequency distribution of mossy fibre rates, and what happens if the input layer of the network is divided functionally into sagittal strips which receive different rates. That is why we vary the shape of the distribution of inputs, to test the effect and the tolerance of the system.

B. Fig 3

Input and output of the granular layer computation are not related by a function. Stated in short form, the input layer of a network receives input from what may be around 80,000 mossy fibres. Inputs are passed through a chain of iterative random samplings (simplified: Golgi cells sample mossy fibre rates and glomeruli sample Golgi cell rates) to calculate inhibition of granule cells at each of 700 glomeruli per field, in 4,100 fields (into which we nominally divide the input layer of the network). Outputs are passed to the granule cell computation to derive the independent outcome of comparing inhibition and randomly sampled excitation of each dendrite of around 9,000 granule cells per field, field by field, to obtain the number that fire in each field, and also the rates they fire at on a normalised scale. The proportion of granule cells which fire is well-predicted by probability, which is controlled by the relationship of inhibition and the mean of mossy fibre rates. Granule cell firing rates are collectively well-predicted by mossy fibre rates, and have a focused range centred on the mean.

A similar statement now appears in section 2. Model parameters are the number of samples and sample size at each step in each field, both usually a range with a distributed probability, typically available (or which we can estimate) from the literature.

The purpose of Figure 3 is to show that in principle the granular layer computation could provide a regulatory mechanism. It is also a purpose to show that the results are untidy, but that they get tidier at low percentages and tidier still in columns 3 and 6, where regulation of the percentage of parallel fibres is very strict because it gains from effectively averaging underlying field percentages. We believe the data as they stand are sufficient to demonstrate those findings.

There is another point here – or perhaps a general statement of the same point. As we understand it, the value of summarising the relationship of data in examples is to distil a generalisation. It is not clear to us that it is possible to boil down a system of the nature we describe to mathematical shorthand, or if you could, that it would be more useful. Parameter space gives us a similar problem – part of the function of the system is to absorb random variables so that it is statistically assured to never use anything like the available state space, even though it is unlikely to ever be in the same state twice.

Comment
Figure 4 Information needs to be better summarized. It would be convenient to show an I-O parameter space.

Response
Please see our response to your comment on Fig 3.

Closing author remark
Thank you for your time and useful comments, which have improved the paper. It is encouraging to hear that some researchers have a similar outlook.

Round 2 reviews
Reviewer 3
Recommendation – Accept after minor revision
Thank you for this paper, it is a very interesting take and is a needed discussion about computational units.

I have a few (small) comments:

4.2 Purkinje cells
- In the discussion of the PCs I am missing a statement about silenced PF-PC synapses.

- It is true that the GrC-interneuron-PC pathway drives a fall in the firing rates of PCs but this has a certain delay. It seems to me that it would be important to mention that an increase in GrC input leads to increase in both PC and interneuron activity which then, with a delay, the interneuron inhibits the activity of the PC.

6. Discussion
- In your final discussion about the cerebellum and microtones I think the review by de Zeeuw 2021 (Bidirectional learning in upbound and downbound microzones of the cerebellum) could help you hypothesise a bit about the existence of differences in microzone computations/functions.

- Further, reading about the synaptic ratios, I thought that maybe the following paper helps your discussion: Voerman et al., 2022, Postsynaptic plasticity of Purkinje cells in mice is determined by molecular identity. It may be the intrinsic differences of the expression profiles of the PCs/microzones that influence and not the the synapse input (or may be ratios in your case).  

Decision letter
Thank for submitting your revised manuscript ID OXFNSC-2024-010.R1 entitled "Computational anatomy: the cerebellar microzone computation". Neither of the initial two reviewers was available, so it has been reviewed by a fresh reviewer. As you will see below, this reviewer has a positive evaluation of your manuscript and has also made some helpful comments. I am therefore inviting you to submit a minor revision, to allow you the opportunity to respond to these comments. Your revised manuscript will be due in 30 days but please let me know if you require an extension. Once I receive your revised manuscript, I will endeavour to make a speedy decision without further review.

Please note that this journal operates with transparent peer review. This means that if your submission is accepted for publication, the full peer review history of your article will publish online alongside your article. This includes reviewer comments, editor decision letters, and your author responses.

To revise your manuscript, log into https://mc.manuscriptcentral.com/oxfnsc and enter your Author Centre, where you will find your manuscript title listed under "Manuscripts with Decisions."  Under "Actions," click on "Create a Revision."  Your manuscript number has been appended to denote a revision.

You may also click the below link to start the revision process (or continue the process if you have already started your revision) for your manuscript. If you use the below link you will not be required to login to ScholarOne Manuscripts.

*** PLEASE NOTE: This is a two-step process. After clicking on the link, you will be directed to a webpage to confirm. ***

https://mc.manuscriptcentral.com/oxfnsc?URL_MASK=51c58d177761430794c2124054c95187

You will be unable to make your revisions on the originally submitted version of the manuscript.  Instead, revise your manuscript using a word processing program and save it on your computer.  Please also highlight the changes to your manuscript within the document by using the track changes mode in MS Word or by using bold or colored text.

Once the revised manuscript is prepared, you can upload it and submit it through your Author Centre.

When submitting your revised manuscript, you will be able to respond to the comments made by the reviewer(s) in the space provided.  You can use this space to document any changes you make to the original manuscript.  In order to expedite the processing of the revised manuscript, please be as specific as possible in your response to the reviewer(s).

IMPORTANT:  Your original files are available to you when you upload your revised manuscript.  Please delete any redundant files before completing the submission.

Because we are trying to facilitate timely publication of manuscripts submitted to the Oxford Open Neuroscience, your revised manuscript should be uploaded within the next 30 days from todays date.  If it is not possible for you to submit your revision in a reasonable amount of time, we may have to consider your paper as a new submission.

Once again, thank you for submitting your manuscript to the Oxford Open Neuroscience and I look forward to receiving your revision.

Authors’ response
OUR RESPONSES (IN SQUARE BRACKETS) ARE INTERLEAVED WITH THE REVIEWER'S COMMENTS.

Thank you for this paper, it is a very interesting take and is a needed discussion about computational units.

RESPONSE [Thank you, and thank you for your helpful comments.]

I have a few (small) comments:

4.2 Purkinje cells

In the discussion of the PCs I am missing a statement about silenced PF-PC synapses.

RESPONSE [We have added a new section, section 4.2.3. This includes a statement that even if the proportion of silent PF-PC synapses is large, the number of signals received at working synapses is still big enough for a single Purkinje cell to receive (at any moment) a representative random sample of parallel fibre rates. That is, a single Purkinje cell receives enough parallel fibre signals at non-silent synapses to ‘read’ the granule cell code. The rest of the discussion of parallel fibre synaptic plasticity remains in the Discussion.]

It is true that the GrC-interneuron-PC pathway drives a fall in the firing rates of PCs but this has a certain delay. It seems to me that it would be important to mention that an increase in GrC input leads to increase in both PC and interneuron activity which then, with a delay, the interneuron inhibits the activity of the PC.

RESPONSE [The new section (4.2.3) also addresses this point. In substance, it argues that the sequence of excitation followed by inhibition is absent in physiological conditions because:

1. Despite granule cells firing in short bursts, the parallel fibre code is collectively analogue at the scale received by a Purkinje cell. Therefore, the sequence observed following a discrete stimulus in experiments disappears. Lag (of disynaptic inhibition behind excitation) is presumably still present, but there is no stepped effect.

2. Lag may be mitigated by basket cells, because they do not contact Purkinje cells dendritically but communicate ephaptically through axonal filaments that enwrap the Purkinje cell soma and first axonal segment. Ephaptic transmission is very fast – virtually instantaneous – so the delay that’s observed in measurements of dendritic inhibition by stellate cells (because there are two steps of synaptic transmission) – is reduced or absent from somatic/axonal inhibition by basket cells (because there is only one step of synaptic transmission).]

6. Discussion

In your final discussion about the cerebellum and microtones I think the review by de Zeeuw 2021 (Bidirectional learning in upbound and downbound microzones of the cerebellum) could help you hypothesise a bit about the existence of differences in microzone computations/functions.

Further, reading about the synaptic ratios, I thought that maybe the following paper helps your discussion: Voerman et al., 2022, Postsynaptic plasticity of Purkinje cells in mice is determined by molecular identity. It may be the intrinsic differences of the expression profiles of the PCs/microzones that influence and not the synapse input (or may be ratios in your case).

RESPONSE [We have added a section to the Discussion about the known effects of EAAT4 on the behaviour of Purkinje cells but not the function of those effects, or why expression has a striped pattern. The reason for not discussing the function of EAAT4-expressing stripes is simply that we do not have a view at this stage. We don’t think we need one to run the arguments we make in the paper. We don’t see a conflict.

It seems clear that expression profiles are functional, or they would not exist. The main purpose of the new section is to explain Purkinje cell expression profiles for a general neuroscience readership. A systems-level understanding of cerebellar function will need to include an explanation of expression profiles, but we are not there yet. We have a grant application in development in which we propose to simulate a neuromuscular system in 4-D as proof of concept of the self-driving swimming model. We hope it will form part of the project to include EAAT4 stripes in the model, but it is an area that needs more work.

Thanks again for your encouraging remarks.

Final Decision letter
It is a pleasure to accept your revised manuscript entitled "Computational anatomy: the cerebellar microzone computation" in its current form for publication in the Oxford Open Neuroscience.  

Please note that this journal operates with transparent peer review. This means that  the full peer review history of your article will publish online alongside your article. This includes reviewer comments, editor decision letters, and your author responses.

Next steps
You will receive an email from no-reply@scipris.com within roughly one week. This is your invitation to sign up for an account with SciPris, Oxford University Press’ author portal hosted by Aptara. You will need to create an account if you do not already hold one. Please register or log into your account and follow the online instructions which will guide you through signing your licence and paying the APC. The email and the portal have clearly signposted support options if you need any help during this process.

Please note that SciPris is a completely different system from ScholarOne, so your credentials to submit your manuscript here will not work there. Once you’ve created a SciPris account, you will be able to use it whenever you publish with Oxford Open Neuroscience or any OUP journal. Please note that OUP will only ever request payment for applicable fees be made via SciPris or to an OUP bank account. If you ever have concerns about the legitimacy of a request, please do not hesitate to contact a customer services agent via the SciPris portal or directly via oupsupport@scipris.com.

Thank you for your fine contribution.  On behalf of the Editors of Oxford Open Neuroscience, we look forward to your continued contributions to the Journal.
